# Searching Data: A Review of Observational Data Retrieval Practices in Selected Disciplines

**DOI:** 10.1002/asi.24165

**Published:** 2019-03-12

**Authors:** Kathleen Gregory, Paul Groth, Helena Cousijn, Andrea Scharnhorst, Sally Wyatt

**Affiliations:** ^1^ Data Archiving and Networked Services, Royal Netherlands Academy of Arts and Sciences Anna van Saksenlaan 51, 2593 HW, The Hague The Netherlands; ^2^ Elsevier Labs Radarweg 29, 1043 NX Amsterdam The Netherlands; ^3^ Elsevier Radarweg 29, 1043 NX Amsterdam The Netherlands; ^4^ DataCite Welfengarten 1B, 30167 Hannover Germany; ^5^ Faculty of Arts and Social Sciences Maastricht University Grote Gracht 82, SZ Maastricht, 6211 The Netherlands; ^6^ Informatics Institute University of Amsterdam, Science Park 904 1098 XH Amsterdam The Netherlands

## Abstract

A cross‐disciplinary examination of the user behaviors involved in seeking and evaluating data is surprisingly absent from the research data discussion. This review explores the data retrieval literature to identify commonalities in how users search for and evaluate observational research data in selected disciplines. Two analytical frameworks, rooted in information retrieval and science and technology studies, are used to identify key similarities in practices as a first step toward developing a model describing data retrieval.

## Introduction

Open research data are touted as having the potential to transform science and fast‐track the development of new knowledge (Gray, 2009). In order for data to fulfill this potential, users must first be able to find the data that they need. This is not a simple task. Facilitating data discovery relies on developing underlying infrastructures, support systems, and data supplies (Borgman, 2015). It is equally important to understand the behaviors involved in data retrieval, but a user‐focused, cross‐disciplinary analysis of data retrieval practices is lacking. This review explores the existing data retrieval literature and identifies commonalities in documented practices among users of observational data as a first step toward creating a model describing how users search for and evaluate research data.

**Table 1:** Users' observational data needs by disciplinary community.

Users in this community…	Need this type of data	For these purposes (*italicized = foreground*, normal = background)
Astronomy	Data from sky surveys, telescopes, archives, repositories, data catalogs, virtual observatory systems	*New questions of old data*, baselines, instrument calibration, physical properties, model inputs, data integration
Earth & Environmental Sciences	Plant, animal, water, weather, solar observations; soil analyses, rock thin‐section and satellite images; maps, geographic, demographic and census data; continuously collected and transmitted data, data at temporal/spatial scales, raw and summarized data	*New questions of old data, meta‐analyses*, calibration, context, baselines, reference, model inputs, verification, comparison, environmental planning, policy‐ and decision making, education, instrument monitoring; data integration
Biomedicine	Images, complete fMRI studies, pathology results, patient observations and demographics; population‐level disease data, behavioral data	*Disease/disorder research, new visualizations*, evaluations, 3‐D anatomical pictures, preparing research outputs, education, patient care
Field Archeology	Field notebooks, photographs, artifacts, stratigraphic baselines; data at temporal/spatial scales	*New insights from data aggregation*, comparison, triangulation; training, dissertations, assignments, preparing tours, inventories of local excavations
Social Sciences	Survey data (often only one question is of interest), long‐running data sets/surveys, interviews, archival documents, images, videos	*Re‐interpret data sets; new questions, comparative research*, comparison, preparations, training, dissertations

**Table 2:** Actions taken to locate data.

Users in this community…	Use these resources	In this way
Astronomy	NASA archives, journals, personal exchanges, personal websites, general search engines	Querying archives, extracting data from articles into new tables, informal personal requests
Earth & Environmental Sciences	Journals, personal exchanges, repositories, databases, natural history collections, general search engines, industry	Extracting data from articles, e‐mail/ telephone/letters, metadata searches, faceted searching, filtering, aggregating data to create new data sets, “bounded” strategies (by journal, location, time)
Biomedicine	Online image repositories, local image and patient information systems, personal image collections, Google Images, journals	local systems—patient name/identifier; Online sources—keyword and hierarchical searches, short queries for images
Field Archeology	Personal connections – museum staff and data producers, natural history collections, museums, repositories/archives, publications	Searching by location (keywords, browsing), collaborations to gain additional data
Social Sciences	Survey banks, data catalogs (that is, DBK), repositories, governmental/ statistical offices, databases, commercial providers, personal connections, publications	Following publication references; survey banks—short queries, mismatch between strategies and database design, DBK—more time spent than in literature searching, keyword searching followed by browsing, filters and author names not used,

**Table 3:** Evaluation criteria with frames used in the literature.

Users in this community…	Use these criteria to evaluate data
Astronomy	**1. Contextual Information**: instrumentation, observational conditions, data processing, original research questions **2. Trust:** author reputation, source reputation
Earth & Environmental Sciences	**1. Contextual Information:** instrumentation, observational conditions, data collection procedures, data processing, provenance, original research questions **2. Quality:** meet community standards, comprehensiveness/continuity over time, estimations and uncertainties, resolution **3. Trust:** source, knowledge of object and data collector, author reputation/affiliation, funder, community membership **4. Understandability:** familiarity with practices, data type, subject; consult experienced researchers, first decode data **5. Ease of access**
Biomedicine	**1. Quality**: noise, resolution, anatomical coverage, image acquisition details **2. Trust:** supporting documentation, social networks **3. Relevance**: experience, combination of textual/visual/medical criteria, visual relevancy, background information, understandability, image quality, modality, source
Field Archeology	**1. Contextual information**: collection methods, instrumentation, observational conditions, provenance, original research goals, baseline geographic/stratigraphic/chronological data **2. Suitability for analysis:** consistent data recording practices **3. Trust**: reputation/affiliation/skill of authors, repository features, language in supporting documentation
Social Sciences	**1. Contextual Information**: collection methods, instrumentation, other analyses, definition/measurement of variables, data handling/processing **2. Quality**: completeness, accessibility, ease of use, credibility, reputation of repository, reputation of author/journal **not** important **3. Relevance:** time frame of study, keywords, citing literature, title and publication year **not** as important **4. Trust**: prior reuse, reputation of data repository, reputation of data producer

Although information retrieval (IR) has been extensively studied for over 60 years (Sanderson & Croft, 2012), data retrieval is a nascent field. Recent studies surrounding the issue examine how data are made available via data sharing (Tenopir et al., 2011, 2015), how researchers reuse data (Faniel, Kriesberg, & Yakel, 2016; Pasquetto, Randles, & Borgman, 2017), and how systems are designed to optimize data discoverability and retrieval (Pallickara, Pallickara, & Zupanski, 2012). Information documenting data retrieval behaviors is buried throughout other disciplinary and data‐related literature and is not easy to identify (Gregory, Cousijn, Groth, Scharnhorst, & Wyatt, 2018).

We draw on work in IR and science and technology studies (STS) to guide the identification of this buried literature and to develop our analysis frameworks. The first framework is based on established models of interactive IR; the second framework builds on STS‐inflected work examining data practices and communities. We begin by discussing the frameworks in more detail before using them to present and synthesize the data retrieval behaviors documented in the collected literature. We end with a discussion of commonalities across disciplinary communities and identify gaps in the literature and areas for future work.

## Framework #1: A Broad View of Interactive IR

IR is an interactive process, involving a dynamic interplay between users and IR systems (Xie, 2008). Numerous models describe user‐oriented interactive IR. Three of the most pivotal are Ingwersen's cognitive model (1992, 1996), Belkin's episode model (1993, 1996), and Saracevic's stratified interaction model (1996, 1997). Detailed characterizations of the strategies (for example, Bates, 1990) and cognitive and affective stages in user‐oriented information seeking (Kuhlthau, 1991) have also been proposed. Despite their differences, established models assume that users are actively involved in the search process and that context influences search behaviors (Rieh & Xie, 2006; Xie, 2008).

Interactive IR models share a few key stages^1^ (Wolfram, 2015) that are used to structure the first framework and to provide the main divisions of this article:

- **Users and Needs**: describes user contexts and data needs.
- **User Actions:** describes the sources and search strategies used to locate research data.
- **Evaluation:** describes criteria and processes used when evaluating data for reuse.

The term “data retrieval” is used in this review to refer to this entire complement of needs, actions, and evaluation behaviors.

## Framework #2: A Broad View of Data Communities

Data practices can define communities in different ways (Birnholtz & Bietz, 2003). Data communities form around disciplinary domains, (Faniel, Kansa, Kansa, Barrera‐Gomez, & Yakel, 2013; Palmer, Cragin, & Hogan, 2004), research approaches and data collection methodologies (Birnholtz & Bietz, 2003; Weller & Monroe‐Gulick, 2014), and particular data sources (Brown, 2003; Sands, Borgman, Wynholds, & Traweek, 2012). Both macrolevel characteristics, such as using quantitative versus qualitative data (Birnholtz & Bietz, 2003) and microlevel characteristics, such as participation in a specific research project (Borgman, Wallis, & Enyedy, 2007) can define community membership. A researcher may belong to multiple data communities simultaneously, or s/he may choose to define his/her community in unique ways (Birnholtz & Bietz, 2003).

Here we embrace a broad approach to conceptualizing data communities. The overarching data community used in this framework is based on accepted classifications of research data. While classifying data is a notoriously difficult task (Borgman, 2015), broad categories that have proven to be useful are observational, experimental, or computational data (National Science Board, 2005; National Science Foundation, 2007). As a first step in testing the validity of this conception of data communities, we focus on a community bounded by the use of a particular data type: observational data.

Observational data result from recognizing, recording, or noting occurrences. They are often produced with the help of instruments, and include weather observations, polling data, photographs, maps, and economic indicators (Borgman, 2015; National Science Board, 2005). Observational data are used across disciplines; we therefore introduce disciplinary communities into the second framework to provide another level of analysis.

STS research explores the role of disciplinary norms and behaviors in data practices (for example, Leonelli, 2016). Subdisciplines and individual research groups may have unique data practices, different from those of the broader disciplinary community (Gregory et al., 2018); while these differences are important, we suggest that commonalities are also important. In order to identify possible commonalities, we group the disciplines represented in the retrieved literature into five broad domains: astronomy, earth and environmental sciences (EES), biomedicine, field archeology, and social sciences.

This review centers on the role of the researcher as data user. While the discussion of data communities often takes the perspective of data producers, researchers play multiple roles, often mixing data production and consumption (Borgman, Van de Sompel, Scharnhorst, van den Berg, & Treloar, 2015). We focus on consumers/users of observational data who use data they did not create either for new purposes and/or to support existing projects.

## Purpose of the Frameworks

Many studies employ case studies, interviews, and ethnographic research to depict particular data practices in fine detail (Cragin, Chao, & Palmer, 2011; Weber, Baker, Thomer, Chao, & Palmer, 2012) and are spread across disciplinary domains. While these studies provide great depth, it is challenging to bring them together in meaningful ways to identify similarities (Faniel, Barrera‐Gomez, Kriesberg, & Yakel, 2013). The primary goal of this review is to use the macroscopic perspectives of the frameworks introduced above to identify commonalities in reported practices. Such a broad approach comes with two drawbacks: the loss of some of the complexity and detail of the original studies and a bias in the disciplinary scope.

Each section begins with a table synthesizing the reviewed literature through the lens of both frameworks. We then present the literature used to create these syntheses, structuring the findings by disciplinary community. In the Discussion, we summarize and discuss the key findings from each section and identify common themes.

## Methods

Our literature collection methodology was informed by the first framework. We performed keyword searches related to IR (for example, user behavior, information seeking) and data practices (for example, data sharing, data reuse, research practices) across all fields, primarily in the Scopus database. We also performed searches related to data search and data discovery and used bibliometric techniques such as citation chaining and related records.^2^

We closely read the nearly 400 retrieved documents to identify articles referring to observational data. As we read, we again applied the first framework, seeking descriptions of data users and their needs, sources, and strategies used to locate data, and the criteria used to evaluate data for potential reuse. Few studies examine data retrieval practices directly; much of the information is buried within investigations of data sharing and data reuse or found in user studies of particular repositories.

## Users and Needs

In this section we analyze the diversity of users' data needs within the context of disciplinary communities, as summarized in Table 1. We adopt the characterization of background uses of data that support research and foreground uses that drive new research (Wynholds, Wallis, Borgman, Sands, & Traweek, 2012).

### *Astronomy*

Much astronomical research can be classified as big science, involving large international projects supported by extensive knowledge‐sharing infrastructures (Borgman et al., 2007). Big science is not the only approach, as astronomers also conduct research falling within the long tail of science (Wynholds, Fearon, Borgman, & Traweek, 2011). Access to the vast amount of available research data is remarkably open, and data sharing is generally encouraged (Hoeppe, 2014; Pepe, Goodman, Muench, Crosas, & Erdmann, 2014).

#### *Data needed*

Data from large‐scale sky surveys, such as the Sloan Digital Sky Survey (SDSS), form the foundation for many research projects (Pepe et al., 2014). Similarly, the data practices of researchers working with the SDSS are the cornerstone of the data retrieval literature in astronomy (Borgman, Darch, Sands, & Golshan, 2016; Sands et al., 2012; Wynholds et al., 2011).

Sky survey data fuel studies involving further data processing; derived data are then used as the basis for publications (Pepe et al., 2014). Direct data from ground‐ and space‐based telescopes, data located in data repositories and catalogs, and data found through federated queries of virtual observatory systems are important sources (Sands et al., 2012; Wynholds et al., 2012). Theoretical researchers also use observational data from established archives as model inputs (Sands et al., 2012).

#### *Data uses*

Astronomers combine multiple data sets, often from multiple archives or telescope types, during a single project (Sands et al., 2012; Wynholds et al., 2011). Merging data about the same target from different instruments poses a significant challenge (Hoeppe, 2014; Zinzi, Capria, Palomba, Giommi, & Antonelli, 2016).

Astronomers use external data for foreground purposes driving new scientific inquiries and leading to new discoveries (Wynholds et al., 2011; Wynholds et al., 2012), and for background purposes supporting research, such as study baselines, calibrating instruments, and searching for specific physical properties (Wynholds et al., 2012).

### *Earth and Environmental Sciences*

A variety of disciplines and subdisciplines are represented in the literature at differing levels of granularity. Data retrieval practices are sparsely documented in fields such as volcanology, but discussions are increasing in other disciplines, such as the water sciences (for example, Dow, Dow, Fitzsimmons, & Materise, 2015). This is partly due to a change in data collection techniques. As researchers transition from primarily manual field work to using sensors enabling continuous collection, they must find new ways to manage their data (Maier et al., 2012). The ecologists involved in the multidisciplinary Center for Embedded Networked Sensing (CENS) are an example of researchers caught in this transition (see Borgman et al., 2007; Wallis, Rolando, & Borgman, 2013).

#### *Data needed*

Biodiversity researchers require an incredible multiplicity of data. Potentially any information about life on earth, from satellite photos to forest inventories, could be important (Bowker, 2000b). Scientists need information about species distribution and occurrence, population trends, and geographic raw data (Davis, Tenopir, Allar, & Frame, 2014). The needs of CENS researchers exemplify what Bowker terms “data diversity,” as they use weather, solar, and river observations, as well as remote sensing and demographic data (Bowker, 2000a; Wallis et al., 2013). Data diversity is also the norm in the geo‐ and water sciences. Volcanologists rely on images of thin rock sections, chemical analyses, and characterizations of the earth's crust. Additionally, stratigraphers use astronomical observations and numerical data extracted from graphs to study geologic history (Weber et al., 2012). Geographers need data spanning the physical and social sciences, requiring topographic, geologic, and demographic maps, satellite images and drawings, and census data (Borgman et al., 2005). Water scientists need streamflow, evaporation, groundwater level, and water quality measurements (Beran, Cox, Valentine, Zaslavsky, & McGee, 2009). Although they do not exist for every condition, continuously collected data that can be analyzed by location and time are expected (Dow et al., 2015).

This need for data at different geographic and temporal scales connects the disciplines. Atmospheric scientists need large amounts of observational data from specific regions and times for their models (Pallickara et al., 2012). Data collected at local levels can be more important than data collected at national or state levels, as shown by a user survey from Davis et al. (2014).

The Davis et al. survey is one of the few that differentiates between the data needs of different types of users; another example is a study from the Center for Coastal Margin Observation and Prediction (CMOP) (Maier et al., 2012). Internal and external researchers using CMOP data want succinct data overviews. Policy and decision makers need thematic collections summarized on one page, with salient data clearly marked; users in education sectors are also interested in CMOP data, although their specific needs have not yet been studied (Maier et al., 2012).

Like researchers, environmental policy and decision makers need information from different locations and times, but they have difficulties accessing the information (McNie, 2007) or finding the right type. Data produced by scientists are not automatically useful for policy makers (Cash et al., 2003). Environmental planners may not need the same depth of information as researchers (Van House, Butler, & Schiff, 1998); reflecting this, differentiated data products for diverse users are being explored (see Baker, Duerr, & Parsons, 2015).

#### *Data uses*

CENS researchers use external data solely for background purposes, such as contextualizing their own data and calibrating instruments (Wallis et al., 2013; Wynholds et al., 2012). Other background uses include benchmarking and as references (Bowker, 2000b). Some ecologists do reuse external data to answer new questions (Zimmerman, 2007) or to create meta‐analyses (Michener, 2015).

Integrating diverse data is problematic across the environmental sciences. Data collected at different scales and using different nomenclatures are difficult to merge (Bowker, 2000b; Dow et al., 2015; Maier, Megler, & Tufte, 2014). Natural variances in systems and populations further complicate fitting biodiversity data together (Bowker, 2000b; Zimmerman, 2007). Stratigraphers use one data set to calibrate another as they construct geologic timelines used as baseline data by other researchers (Weber et al., 2012). Atmospheric scientists and climatologists grapple with problems stemming from metadata variation (Pallickara et al., 2012) and differences in community data practices (Edwards, Mayernik, Batcheller, Bowker, & Borgman, 2011).

Modelers use external data at specific points in the research process. After reformatting and regridding data to fit model specifications, earth scientists use observational data to initially force models and for parameterization; data availability limits the types of studies undertaken (Parsons, 2011). Coastal modelers engage in similar behavior, continually calibrating and benchmarking their models, and comparing outputs to external observational data (Maier et al., 2012; Weber et al., 2012).

Environmental planners use data not only to make decisions, but also to defend their viewpoints, to persuade, and in education (Van House et al., 1998). Although detailed studies of nonscientists' data needs are lacking (Faniel & Zimmerman, 2011), reported “background uses” of oceanographic data include preparation for triathlons, search and rescue operations, or fishing expeditions (Weber et al., 2012).

### *Biomedicine*

The biomedical literature focuses on fields centering on imaging, such as neuroscience and radiology.

#### *Data needed*

As neuroscience embraces big science methodologies, the field is struggling with how to make data available, discoverable, and usable (Choudhury, Fishman, McGowan, & Juengst, 2014). Researchers rely on visualizations of normal and abnormal brains, although they also consult brain bank samples (Beaulieu, 2004). Sometimes researchers need raw functional magnetic resonance imaging (fMRI) studies, including detailed metadata; sometimes images and scans suffice (Key Perspectives, 2010; Van Horn & Gazzaniga, 2013). Neuroimaging data are complex, consisting of numerous brain section slices, timepoints, and other variables (Honor, Haselgrove, Frazier, & Kennedy, 2016). Neuroscientists incorporate more than just imaging into their work, using demographic, genetic, and behavioral data (Williams et al., 2009).

Clinicians and medical researchers also use a mixture of images and other observational data, such as pathology results, clinical data (for example, progression of tumor grades), patient demographics, and population‐level disease data (Kim & Gilbertson, 2007). Medical images are an essential part of workflows in fields such as radiology (Markonis et al., 2012), where healthcare professionals tend to search for two types of images: general medical images (for example, images of anatomic organs) and specific medical images, which are used for clinical or comparison purposes (Sedghi, Sanderson, & Clough, 2011). Users need images collected with different modalities (X‐rays, computed tomography [CT] scans, and MRIs) (Kim & Gilbertson, 2007); medical students need images corresponding to their current courses (Müller et al., 2006). All reusable medical data must be provided in a way protecting patient privacy (Erinjeri, Picus, Prior, Rubin, & Koppel, 2009).

#### *Data uses*

Neuroscientists use imaging data for comparisons, evaluations, and creating 3D pictures of brain anatomy (Beaulieu, 2004). A single scan is of little value unless incorporated into a larger database of scans. Aggregating individual scans creates complete virtual brains that can be manipulated to facilitate new discoveries (Beaulieu, 2004), as in the case of combining fMRI scans from different populations to yield insights about Alzheimer's biomarkers. (Van Horn & Gazzaniga, 2013).

In a study of clinicians, researchers, educators, librarians, and students, users incorporate images in research, patient care, and education (Hersh, Müller, Gorman, & Jensen, 2005). A follow‐up study further characterizes these needs, showing that images are used for self‐education; educating medical students, patient education, making difficult diagnoses; and developing research ideas, grant proposals, and publications (Kalpathy‐Cramer et al., 2015).

### *Field Archeology*

Archeology is another field in transition. Methodologies and data practices are changing, as data move away from being published in analog‐only formats to being made available in digital repositories (for example, Arbuckle et al., 2014); this facilitates data aggregation to study phenomena such as domestic livestock expansion (Arbuckle et al., 2014; Atici, Pilaar Birch, & Erdoğu, 2017). Interdisciplinarity and data diversity are thriving in archeology, as research projects can involve soil scientists, zooarchaeologists, and material scientists (Faniel, Kansa, et al., 2013).

Metadata and documentation of methods and site conditions are extremely important in archeology, as original sites are often “decomposed” during the research process (Faniel, Kansa, et al., 2013). Data recording and metadata standards do not exist (Faniel & Yakel, 2017), making integration across contexts and collection methodologies challenging (Faniel & Yakel, 2017; Niccolucci & Richards, 2013).

Field archeologists need field notes, photographs, and artifacts in museum collections (Faniel, Kansa, et al., 2013). Geographic, stratigraphic, and chronological baseline data are also vital (Atici, Kansa, Lev‐Tov, & Kansa, 2013). Archeologists compare finds from the field to museum collections, often triangulating data from multiple sources (Faniel, Kansa, et al., 2013). Researchers are not the only “consumers” of archeological data: students, hobbyists, and employees of museums and companies use data for diverse background and fewer foreground purposes, for example, aggregating discrete units of “raw data” (Borgman, Scharnhorst, & Golshan, forthcoming).

### *Social Sciences*

Reusing quantitative data in the social sciences is well established (Faniel & Yakel, 2017; Kriesberg, Frank, Faniel, & Yakel, 2013); the reuse of qualitative data is complicated by issues of participant confidentiality and the embeddedness of the researcher in data creation (Broom, Cheshire, & Emmison, 2009).

Social scientists need data from surveys and long‐running data sets (Shen, 2007). Researchers are often interested in only one data point or survey question. Details about the operationalized variables or measured constructs usually are not present when examining individual questions in isolation (Dulisch, Kempf, & Schaer, 2015). Social scientists also need archival documents, images, videos, and interview data (Karcher, Kirilova, & Weber, 2016).

Data can be reused for comparative research or to ask new questions, reinterpret data sets, or verify findings (Corti, 2007). Background uses, that is, preparing for data collection, are common (Parry & Mauthner, 2005).

Kriesberg et al. examined the needs of early career researchers (ECRs) in quantitative social sciences, archeology, and zoology. External data are used in training and dissertations; young researchers may reuse data more often, due to difficulties collecting their own data (2013).

## User Actions

This section examines the resources and strategies used within different communities to locate data (see Table 2).

### *Astronomy*

Astronomers are generally efficient information seekers, in part due to strong disciplinary infrastructures and tools (Meyer et al., 2011). SDSS users download data directly from NASA archives or obtain them from public data releases (Sands et al., 2012). Discovering and tracking down smaller data sets is challenging; SDSS users sometimes browse personal websites or use general search engines. They then contact research groups directly with their data requests. Despite well‐developed infrastructures, personal networks remain an important means for identifying and obtaining data (Sands et al., 2012).

Journal articles are another important data source. Astronomers copy and paste or transcribe data from articles into new tables for further manipulation (Pepe et al., 2014). Direct citation of archival accession numbers facilitates data discovery from journals (Swan & Brown, 2008).

### *Earth and Environmental Sciences*

Finding and accessing biodiversity data can be challenging, although academics have an easier time than government employees and program managers. A lack of training, time, and knowing where to look hinders effective data search among these groups (Davis et al., 2014). Knowing where to search can be especially problematic in areas outside of a researcher's primary expertise (Devarakonda, Palanisamy, Green, & Wilson, 2011) and is contingent on knowing that data even exist (Zimmerman, 2003). Personal experiences with data collection and a familiarity with research trends help researchers estimate whether data are extant and findable (Zimmerman, 2007).

Compounding this problem, data are distributed across numerous repositories (Dow et al., 2015). Users must first discover the repository, and then invest significant time and energy becoming familiar with each search environment (Ames et al., 2012; Beran et al., 2009). Given the diversity of interfaces, it is not surprising that water scientists desire a “Google for data” (Megler & Maier, 2012).

In a global survey of the environmental research community, the majority of respondents discover data through journal articles, search engines, and disciplinary repositories; 40% request data directly from data providers (Schmidt, Gemeinholzer, & Treloar, 2016). Although some environmental planners are interested in using journals and primary sources, they find it too time‐consuming (Miller et al., 2009), and may instead turn to colleagues for biodiversity information (Janse, 2006; Pullin, Knight, Stone, & Charman, 2004).

Stratigraphers extract data from journals, laboriously recreating tables from published graphs. They are willing to spend money as well as time obtaining data, sometimes purchasing expensive high‐resolution data from drilling companies (Weber et al., 2012). Geographers utilize journals and search engines to locate maps, images, and repositories, but poor indexing and metadata derail their efforts (Borgman et al., 2005). Ecologists in Zimmerman's studies gather single data points from multiple sources and then aggregate them to create new data sets (2007; 2008), an approach that is increasingly common in biodiversity research (Davis et al., 2014).

Personal exchanges are valuable, if complex, sources of external data. Requesting data from CENS, for example, is a multistep process. Data seekers identify CENS as a potential source, contact the CENS researcher, and discuss the availability and suitability of the data. The CENS researcher then gathers, processes, and delivers the requested data (Wallis et al., 2013). Ecologists employ a variety of tactics (e‐mails letters, and telephone calls) to obtain data mentioned in articles. As organizations grow and such requests increase, personal exchanges cease to be an effective way to obtain data (Wallis et al., 2007).

Ecologists reusing data employ “bounding” strategies, limiting searches to particular journals, times, or locations to collect representative samples (Zimmerman, 2007). As data seeking is data collection, these researchers use strategies that minimize error, can be publicly defended, and increase the likelihood of accessing data (Zimmerman, 2007). They have specific search criteria; the general information in databases usually does not meet their detailed needs (Zimmerman, 2007). Before building specific search tools, CMOP researchers struggled with similar problems, retrieving either zero or thousands of hits. If researchers found searching too frustrating, they would simply stop searching (Maier et al., 2012; Megler & Maier, 2012).

Large atmospheric data sets, encoded in binary formats to facilitate storage and transfer, cannot effectively be searched with text‐based search engines. Rather, users must browse collections using metadata schemas (Pallickara, Pallickara, Zupanski, & Sullivan, 2010). For other data, that is, data sets in the DataONE platform, users prefer keyword searches, followed by filtering (Murillo, 2014).

### *Biomedicine*

While it has become easier to locate data, for example in neuroscience (Beaulieu, 2004), access restrictions still frustrate researchers (Honor et al., 2016).

Medical image retrieval studies show that users search both local restricted‐access systems and free Internet sources. Local systems, including Picture Archiving and Communication Systems (PACS), electronic patient records, hospital archives, and teaching files, house images and patient data (Müller et al., 2006). Radiologists also curate their own collections of images stored on personal computers (Markonis et al., 2012).

Despite access to specialized collections, Internet searches, particularly with Google Images, are common (Markonis et al., 2012; Müller et al., 2006). Limitations of such searches include sifting through irrelevant results and a dearth of highly‐specialized images. Nevertheless, online image repositories are unpopular among healthcare professionals, perhaps because of their limited scope (Sedghi et al., 2011). Academic journals, however, facilitate locating specialized, cutting‐edge images with contextual information that is difficult to locate on the web (Sedghi et al., 2011).

Search strategies vary depending on the searcher's professional role, although commonalities do exist. Users often search by patient names or identifier in PACS for diagnostic purposes; brief keyword or hierarchical searching is typical in nondiagnostic searching (De‐Arteaga et al., 2015; Markonis et al., 2012; Müller et al., 2006).

Success is not assured when searching for images. In a study of radiologists, users fail to find desired images in almost 25% of cases. Users believe these images exist, but that they simply cannot be found (Markonis et al., 2012). Possible search difficulties stem from a lack of time and available relevant articles, the newness of certain topics, and a lack of domain‐specific search tools (Sedghi et al., 2011).

### *Field Archeology*

Data discovery is a significant problem in field archeology. Data are scattered among collections or sometimes are only in unpublished field reports (Niccolucci & Richards, 2013). Although publications are used in data discovery (Faniel & Yakel, 2017), they do not consistently include data; a significant delay between data collection and publication exacerbates the problem (Kriesberg et al., 2013). Researchers often do not know what data are available (Aloia et al., 2017). ECRs circumnavigate difficulties by collaborating with supervisors to locate data (Kriesberg et al., 2013). Other archeologists turn to personal networks, museums, and, as the shift toward digital data continues, data archives (Faniel, Kansa, et al., 2013; Faniel & Yakel, 2017). Details about how users search archives are sparse (Borgman et al., 2015), although searching and browsing by location are important strategies often complicated by differences in geographic terminology (Borgman et al., forthcoming).

### *Social Sciences*

Social scientists use data from governmental/statistical offices and specialized databases (Shen, 2007). Economists also obtain data from statistical offices but may purchase data directly from commercial providers (Bahls & Tochtermann, 2013). Researchers easily locate data from national, publicly funded data sets, but struggle to locate smaller data sets and video data for reuse (Key Perspectives, 2010). Researchers tap publications or make direct requests to find these more specialized data (Swan & Brown, 2008).

Personal networks, including advisors, coworkers of advisors, or former employers are key sources of qualitative data (Yoon, 2014b), especially for ECRs, who rely on journal recommendations from advisors and observations of their colleagues (Faniel & Yakel, 2017; Kriesberg et al., 2013). Not knowing whom to contact or where to begin searching makes locating relevant data difficult (Curty, 2016).

Searchers of the DBK, the primary catalog for social science data in Germany, expend more time and effort when seeking data sets than they do for publications. These researchers do not frequently use author names; rather, keyword searching, followed by browsing long results lists, are more frequent strategies. Researchers complain about a lack of filtering options, but do not use available filters (Kern & Mathiak, 2015). Social scientists search a survey bank by short keyword queries or social construct, even though these strategies do not match the database's structure (Dulisch et al., 2015).

## Evaluation

We identify major frames used in the literature to discuss data evaluation criteria, including trust, quality, necessary contextual information, and relevance. The frames overlap, as the characteristics composing these frames vary from article to article, both within and across disciplines. In Table 3, we present the evaluation criteria and associated frames as they are discussed in the literature.

### *Astronomy*

Astronomers rely on detailed documentation of instrumentation, collection methods, and conditions, data processing, and original research questions (Borgman et al., 2016; Wynholds et al., 2011). They know which authors to trust and believe data in NASA archives and established projects are valid, accurate, and trustworthy. Researchers must completely understand data and the creation processes; they would rather recreate data before using poorly documented secondary data products (Wynholds et al., 2011).

### *Earth and Environmental Sciences*

When evaluating data for reuse, researchers use contextual information about data provenance (Dow et al., 2015; Murillo, 2014), technical instrumentation (Wallis et al., 2007), and original research questions (Zimmerman, 2008). Researchers reuse data they understand, seeking data collected via practices they have used themselves (Zimmerman, 2007, 2008) and with familiar data types (Murillo, 2014). Contextual details are found in field notebooks (Weber et al., 2012) and articles (Carlson & Stowel‐Bracke, 2013), but additional metadata attached to data sets are the preferred method of conveying context (Bowker, 2000b). Formal metadata has limitations, however, as they cannot always contain enough detail or inspire the confidence needed for reuse. Researchers may instead base decisions on the word‐of‐mouth reputation of the data set (Weber et al., 2012) or rely on more experienced researchers to develop understanding or alternative evaluation strategies (Zimmerman, 2008).

Data must have sufficient quality, often defined by community standards, to be reused (Zimmerman, 2007). Water researchers and earth science modelers consider comprehensiveness and continuity over time and space (Dow et al., 2015; Parsons, 2011) as well as uncertainties and error estimates (Larsen, Hamilton, Lucido, Garner, & Young, 2016; Parsons, 2011) when determining data quality. Volcanologists use image resolution as a quality indicator (Weber et al., 2012).

Ecologists trust data from well‐known sources, such as databases and literature (Zimmerman, 2007), and make decisions based on authors' reputations and affiliations (Murillo, 2014; Weber et al., 2012). Initial evaluations are based on the reputation of the source where the data were discovered, even if researchers eventually obtain them through other means (Zimmerman, 2007). Standardized collection practices are not enough to establish trust, as practices themselves say nothing about the data collector's skill (Zimmerman, 2008). The sponsor of research (McNie, 2007) and membership in the same community of practice (Van House et al., 1998) facilitate trust among environmental planners and policy makers.

Both ecologists and modelers reuse data that are easy to access (Parsons, 2011; Zimmerman, 2007). Modelers, however, face an extra step in the evaluation process, needing first to decode numerically encoded data sets before deciding if they are appropriate (Pallickara et al., 2010).

### *Biomedicine*

Visual, medical, and textual criteria are used to evaluate biomedical images. Healthcare workers rank visual relevance, background information, and image quality as being most important, although they also mention image modality and understandability (Clough, Sedghi, & Sanderson, 2008). Radiologists rely on a mixture of image properties, image quality, supporting documentation, and information about the source to determine suitability (Markonis et al., 2012).

Evaluation criteria vary depending on users' professional specialties and particular situations (Clough et al., 2008). Users rely on visual attributes when evaluating general medical images but incorporate textual information and credibility criteria for specific images used for background purposes (Sedghi et al., 2011).

Definitions of quality also vary by user. A neurosurgeon, for example, uses noise levels, resolution, and anatomical coverage, while a radiologist focuses mostly on motion artifacts to determine image quality (Heckel, Arlt, Geisler, Zidowitz, & Neumuth, 2016). Resolution and acquisition details (for example, slice thickness in tomographic images) are other proxies for quality (Müller et al., 2006).

Healthcare professionals determine relevance through a combination of textual background information, visual inspection, and mental comparison to imagined ideals (Sedghi, Sanderson, & Clough, 2012). Personal experience trumps other criteria, however, when determining image relevance (Markonis et al., 2012; Müller et al., 2006).

Clinicians build trust in images through supporting documentation, such as attached exams or biopsies. Systems allowing researchers to comment on images online can also build trust normally created through informal “hallway” communications (Jirotka et al., 2005; Markonis et al., 2012).

### *Field Archeology*

Archeologists require contextual information about collection methods, instrumentation, observational conditions, and artifact provenance (Faniel, Barrera‐Gomez, et al., 2013). Other fundamental metadata include information about original research goals and baseline geographic, stratigraphic, and chronological data (Atici et al., 2013). Current metadata schemas are not rich enough to provide this level of contextual description. Archeologists either make do with the available information or seek other ways to further develop context (Faniel, Kansa, et al., 2013).

Consistent data recording practices (for example, an absence of misspellings or translational errors) (Atici et al., 2013), and detailed language in supporting documentation (Faniel, Kansa, et al., 2013) help to establish credibility and trustworthiness. Author reputation and affiliation and repository features, such as metadata type and level of transparency, help to establish trust (Faniel, Kansa, et al., 2013).

### *Social Sciences*

DBK users spend more time evaluating data results compared with literature results, consulting additional documentation when needed. Researchers appear to think this is normal, perhaps because choosing the correct data set is more important than selecting the correct article (Kern & Mathiak, 2015). Title and publication year are not as important as study time frame and keywords in evaluations. Users would like access to literature citing a data set to determine if a research question has already been answered (Kern & Mathiak, 2015); prior reuse of data is also an important way of developing trust (Faniel & Yakel, 2017).

Data seekers rank accessibility as the most important factor determining satisfaction with data reuse in the ICPSR repository. Data completeness (ranked 2nd), credibility (4th), and ease of use (5th) are also contributing factors; in this study, journal/author reputation do not appear to impact satisfaction (Faniel et al., 2016). Other work suggests that the repository reputation is an important signal of data quality and credibility (Curty, 2016) and is used to develop trust in data (Faniel & Yakel, 2017). Data reusers tend to either make do with available data or adapt their research projects to use data that they can find. The more researchers have to “reshape” their projects, the less satisfied they are (Faniel et al., 2016).

Users need contextual information about collection methods, instrumentation, other analyses, and how variables are defined and measured (Curty, 2016; Faniel, Kansa, et al., 2013; Kern & Mathiak, 2015; Yoon, 2014a). When necessary, researchers turn to other sources to develop the necessary context (Fielding & Fielding, 2008), consulting colleagues, codebooks (Faniel & Yakel, 2017), or bibliographies (Faniel, Barrera‐Gomez, et al., 2013). Ideally, specialized metadata schemas would provide enhanced context (Kern & Mathiak, 2015). Debate remains, however, if documentation can build the context needed to reuse qualitative social science data (Broom et al., 2009; Parry & Mauthner, 2005).

Novice researchers especially need supporting contextual information. They want details about coding procedures, collection methods, and data set merging and matching (Faniel, Kriesberg, & Yakel, 2012). More experienced researchers can make do more easily with limited documentation (Yoon, 2016).

## Discussion

Having presented the documented practices of observational data users, we use the frameworks to synthesize our key findings and to identify commonalities and themes spanning the reviewed disciplinary communities.

### *Users and Needs*

Researchers across and within the reviewed disciplines need a diversity of observational data, requiring data of different types from different sources and disciplines, collected at different scales using different instruments. Users have very specific requirements, needing data from particular locations (geographic, anatomical, or astronomical), at particular resolutions or collected using particular mechanical or survey instruments.

Integrating diverse data is necessary but challenging. Astronomers struggle to bring together data from different telescopes, neuroscientists try to combine neuroimages with clinical data, and archeologists need to integrate data collected in different contexts with different methodologies. Some of these challenges may be augmented by changes in research practices, such as automated data collection in EES (Borgman et al., 2007), or by shifts in community data practices, such as increased data sharing, as in archeology (Arbuckle et al., 2014) or neuroscience (Choudhury et al., 2014).

Background and foreground uses are reported across disciplines, although background uses are better documented. These include making comparisons, benchmarking, preparing research projects, calibrating instruments, and as model inputs. Reported foreground uses are vaguer, often limited to reports of “asking new questions of data.” This does not mean that foreground uses do not occur; examples of new research fueled by data reuse could likely be found in all of the reviewed disciplines (for example, Atici et al., 2017). This could indicate a mismatch between what studies of data practices report and actual practices, or it could be a sign of changing practices. Even with a broad analysis, we see that data use varies within disciplines. One group of biodiversity researchers uses secondary data only to support projects, for example, while another study only examines cases of foreground use. Other possible data uses, that is, in teaching, clinical practice, or environmental planning, are hinted at, although rarely explored in detail.

A generic view of the user is also common. Similar to our approach, disciplines are often broadly represented; the social sciences in particular tend to be treated as a homogenous group. Few studies document the needs and behaviors of specific user groups, such as early career researchers (Kriesberg et al., 2013; Faniel et al., 2012), policy makers (Janse, 2006; McNie, 2007; Cash et al., 2003) or students (Carlson & Stowel‐Bracke, 2013). Understanding the data practices of ECRs sheds light on processes of acculturation (Kriesberg et al., 2013) and is important, as large‐scale data reuse depends on adoption by ECRs (Faniel et al., 2012). Understanding the practices of specific user groups is also critical in designing user‐oriented data discovery systems.

### *User Actions*

Across communities, users find data in repositories, journals, on websites, and through personal networks. This variety could be due to differing infrastructures available within disciplines; however, even in fields with established data repositories, that is, astronomy and quantitative social science, researchers seek data outside of these systems (Faniel & Yakel, 2017; Sands et al., 2012).

Personal exchanges are valuable sources of external data. While locating large, well‐known data sets is straightforward, tracking down smaller, specialized data sets is challenging and often requires personal communication (Sands et al., 2012). Existing repository search functionalities may not meet the specific needs of researchers, or users may not develop appropriate search strategies in these resources (Sedghi et al., 2011). Users may also simply not be aware of the existence of data or databases; this may be especially true for researchers seeking data outside of their primary disciplines.

The distributed nature of observational data compounds these problems. A variety of data repositories exist within these disciplines (for example, Dow et al., 2015); within each new resource, users must start from scratch—first discovering the resource, then investing significant time and energy becoming familiar with it and the available data. A lack of time and accessible data also complicates the search process.

### *Evaluation*

Researchers across disciplines need as much contextual information as possible, requiring documentation about instruments, methodologies, research questions, and observational conditions. This information is combined with the reputation of the repository and often that of the data author to establish trust, data quality, and relevance. Although much of the reviewed literature uses frames such as trust and quality to discuss evaluation, the characteristics used to develop these frames varies. This variation may result from disciplinary or individual differences or from how the articles' authors define these frames. One commonality that we can identify is the association of more social criteria—such as the reputation of authors and data sources—in developing trust.

Enriched metadata are often the desired, although imperfect, methods of conveying contextual information. Perhaps because of limitations in metadata, researchers build the needed information by combining a variety of sources, from codebooks and academic literature to unpublished reports and museum records (Faniel & Yakel, 2017). Researchers across communities also use social connections and personal exchanges to evaluate data. The discussion about how researchers evaluate data is still developing, although the process seems to differ from how researchers evaluate academic literature.

The following themes bridging both frameworks emerge from this synthesis:

- A tension between breadth and specificity.
- The social aspects of data retrieval.
- Absent practices and communities.

When developing the frameworks for this article, we presented the tension involved in applying broad perspectives to understand individual practices. This tension between breadth and specificity is also present in the reviewed data retrieval practices. Even within disciplines, researchers need a diversity of observational data and employ a wide variety of search and evaluation strategies. At the same time, users seek data with very precise characteristics. They appear to balance breadth and specificity as they work to integrate data sets from diverse sources to meet specific needs or to piece together a variety of evaluation criteria to make decisions about reuse.

Social connections and personal exchanges permeate observational data retrieval. Users rely on personal connections and their own networks to locate, obtain, and evaluate data, even in disciplines with extensive infrastructures. This suggests that it is not enough to understand data retrieval as a series of interactions between users and search systems; rather, data retrieval is in fact a complex sociotechnical process.

The absence of many communities and practices in the literature is also apparent. A relatively small number of disciplines are represented in our literature corpus. Among the broad disciplinary categories that we employ, certain subdisciplines are well represented; others are briefly mentioned, and others are treated homogeneously. Building a robust picture of observational data retrieval requires a deeper understanding of practices in other disciplines and of understudied user groups such as nonscientists or early career researchers. Deeper studies of how data retrieval practices change when seeking data for foreground purposes, or when seeking data from different disciplines, are also absent. Although Faniel and Yakel (2017) have recently identified five “trust markers” important in data reuse in archeology, social sciences and zoology, common frameworks for discussing evaluation criteria across the observational data community are lacking.

## Conclusion: Toward a Model for Data Retrieval

Through our analysis we have achieved the following:

- Shown that a framework based on interactive IR is applicable to understanding the data retrieval literature.
- Tested the boundaries of defining data communities, using broad classifications to identify commonalities in practices.
- Revealed absent practices and highlighted areas where more research is necessary.
- Suggested that a framework based on IR alone is insufficient for completely understanding the complexity of data retrieval practices.

The literature also points to ways that IR and data retrieval differ. Data needs are specific, requiring high precision in IR systems (Stempfhuber & Zapilko, 2009). Textual queries and ranking algorithms do not work well for retrieving numeric or encoded data (Pallickara et al., 2010). Users employ different search strategies when seeking data rather than literature (Kern & Mathiak, 2015) and take different roles when interacting with data repositories (for example, as consumers and creators), which can impact system design (Borgman et al., 2015). Researchers also spend more time evaluating data sets (Kern & Mathiak, 2015), perhaps because lists of data cannot be efficiently evaluated in the same way as document lists (Kunze & Auer, 2013).

These differences, in conjunction with the themes identified in the Discussion, suggest that current IR models may not completely describe data retrieval practices. Identifying commonalities in observational data retrieval practices is a first step in exploring possible characteristics of a new model for data IR. Further studies of different data communities, such as users of experimental and computational data, big and long‐tail data seekers, and members of underrepresented user groups are needed. A model describing data retrieval would provide insight into the needs and practices of users that could be applied to both systems design and policy developments for facilitating data discovery.

## Footnotes

1 IR systems are also an important part of these models. The first version of the article preprint (https://arxiv.org/abs/1707.06937) includes an additional review of data retrieval systems.

2 For a detailed methodology and machine‐readable bibliography, including references regarding data retrieval systems, see: https://doi.org/10.17026/dans-zgu-qfpj
